# EBM BLS: Tirzepatide Helps Obese Patients Lose Weight

**DOI:** 10.1007/s11606-025-09815-w

**Published:** 2025-09-03

**Authors:** Hugo Ortega, Harry B. Burke

**Affiliations:** 1 DipABLM Albert Einstein College of Medicine, Montefiore Medical Center, Bronx, NY USA; 2 Uniformed Services University of the Health Sciences, Bethesda, MD USA

**Tip for patients:** For patients who are obese, Tirzepatide provides significant sustained weight loss.

**Source Article:** Jastreboff AM, Aronne LJ, Ahmad NN, et al. Tirzepatide Once Weekly for the Treatment of Obesity. N Engl J Med. 2022;387(3):205-216. doi:10.1056/NEJMoa2206038

## WHY THIS IS IMPORTANT

**Figure 1:**
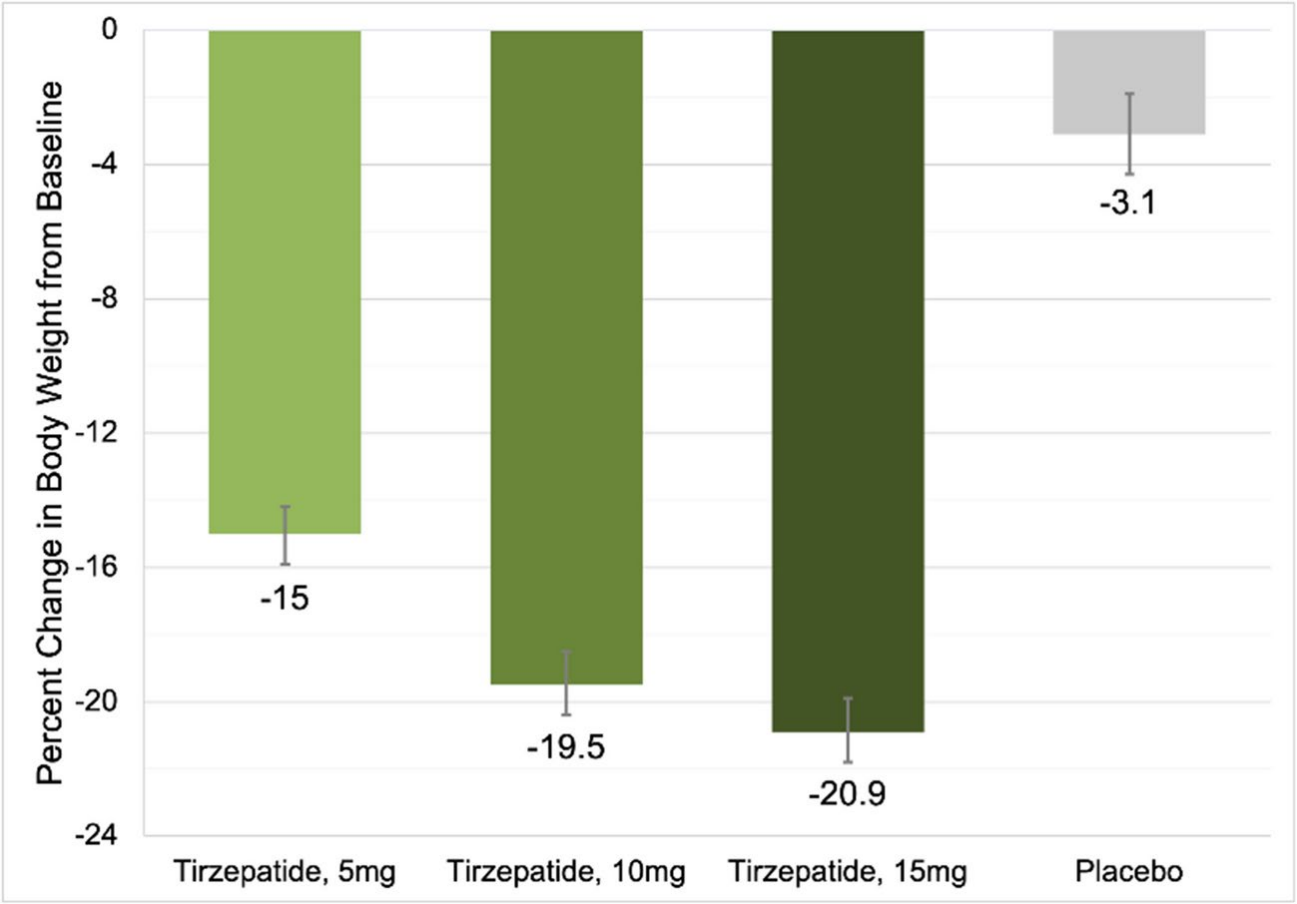
Dose-based effect of tirzepatide on body weight.

- Obesity is a risk factor for many chronic conditions including cardiovascular death.^2^
- Obesity has been on the rise amongst the American population with a prevalence of 41.7% in 2017.^3^ This is up from 30.5% in 2000.^3^ The estimated annual cost of obesity is around $173 billion with medical costs being $1861 higher yearly than for people at a healthy weight.^4^
- Tirzepatide is a dual Glucagon Peptide Like 1 (GLP1) and Glucose-dependent Insulinotropic polypeptide (GIP) agonist. Mimicking effects of naturally produced incretins which stimulate insulin secretion, decrease glucagon production, and suppress appetite (Fig. 1).

## RESULTS

- 2539 patients were enrolled in the trial. 70.6% of the patients were white, 67.5% were female, mean age was 44.9 years old, and the average BMI was 38 kg/m^2^. 40.6% had prediabetes.
- Average changes in body weight percent were as follows: − 3.1% placebo, − 15% (35.5 lbs) 5 mg tirzepatide, − 19.5%(48.9 lbs) 10 mg tirzepatide, and − 20.9% (52.0 lbs) 15 mg tirzepatide.
- Weight loss of 20% or more occurred in over 50% of patients in the 10 mg and 15 mg groups. The weight loss effect of the medication plateaued before 72 weeks.
- Patients on tirzepatide had a decrease in blood pressure (− 7.2 mmHg), triglycerides (− 24.8%), and fasting insulin levels (− 42.9%).
- The most common side effects were related to gastrointestinal effects. Nausea (24.6%, 33.3%, 31%) and diarrhea (18.7%, 21.2%, 23%) in 5, 10, and 15 mg groups respectively.
- Serious adverse events occurred in 6.8% of placebo, 6.3% of 5 mg, 6.9% of 10 mg, and 6.2% of 15 mg groups with 4 reported cases of pancreatitis. Risk of hypoglycemia was ~ 1% higher in the tirzepatide group.

## STUDY DESCRIPTION

### Setting

- This was a phase 3 multicenter, double-blind, randomized placebo-controlled study. The study was conducted at 119 sites across nine countries. Patients aged 18 + with BMI > 30 or BMI > 27 with one or more weight related complications (hypertension, dyslipidemia, cardiovascular disease, obstructive sleep apnea) with reported unsuccessful dietary intervention were included.

### Exclusion Criteria

- Patients with diabetes, recent weight loss of > 5 kg, previous/planned surgical intervention, or on anti-obesity medications were excluded.

### Intervention

- Patients randomly assigned to 1 of 4 groups Tirzepatide 5 mg,10 mg, 15 mg, or placebo.
- Treatment with once weekly injections lasted for 72 weeks.
- All groups were paired with lifestyle counseling sessions by a dietician or healthcare professional. The sessions promoted balanced meals, 500 cal deficits, and 150 min of physical activity weekly.

## Study Quality and Application for Patients

- The study quality was good with minimal oversight from pharmacy company sponsoring the drug. Participants including placebo all received similar lifestyle quality interventions to minimize confounding.
- Of note the study started in Dec 2019, as such COVID-19 may have inflated the number of adverse reactions.
- As a phase 3 trial, this trial cannot fully explore all potential risks and adverse events associated with this medication
- For patients who are overweight (BMI 25–30), results may be different as only a small number of patients (5.5%) were overweight in this study.
- Those who signed up for this study may be more motivated to lose weight than the general population and so results may be smaller in those who do not apply healthy lifestyle changes or have similar access to the education provided to the trial participants.
- To maximize benefits of tirzepatide, it is essential to continue aiming for 150 min of exercise per week, ensure balanced meals, and achieve a calorie deficit.
